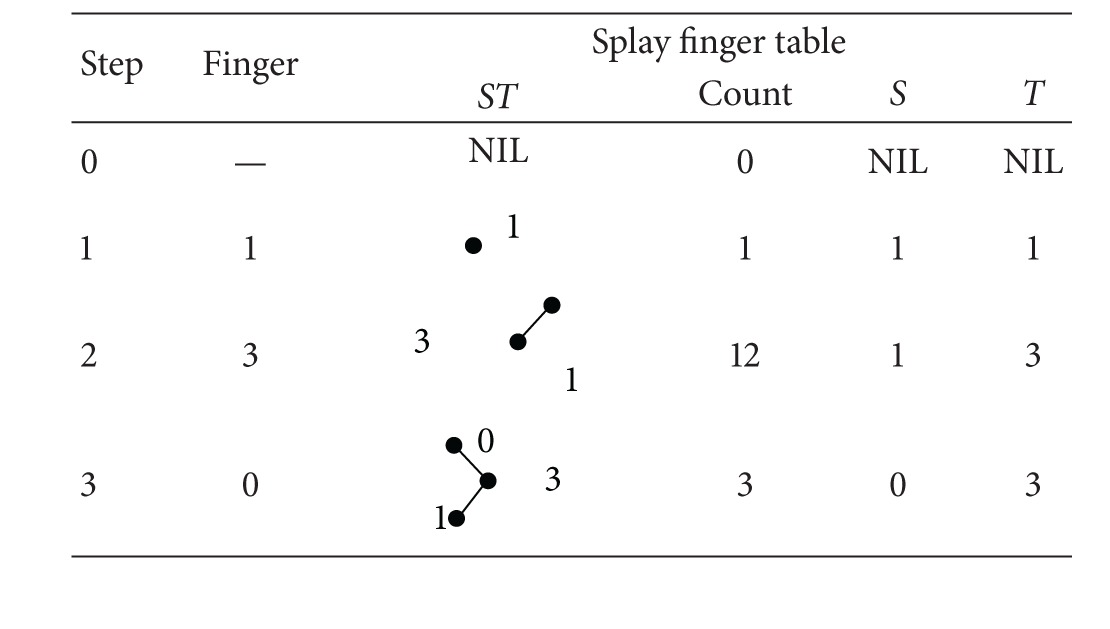# A Splay Tree-Based Approach for Efficient Resource Location in P2P Networks

**DOI:** 10.1155/2014/830682

**Published:** 2014-03-11

**Authors:** Wei Zhou, Zilong Tan, Shaowen Yao, Shipu Wang

**Affiliations:** National Pilot School of Software, Yunnan University, Kunming City 650091, China

## Abstract

Resource location in structured P2P system has a critical influence on the system performance. Existing analytical studies of Chord protocol have shown some potential improvements in performance. In this paper a splay tree-based new Chord structure called SChord is proposed to improve the efficiency of locating resources. We consider a novel implementation of the Chord finger table (routing table) based on the splay tree. This approach extends the Chord finger table with additional routing entries. Adaptive routing algorithm is proposed for implementation, and it can be shown that hop count is significantly minimized without introducing any other protocol overheads. We analyze the hop count of the adaptive routing algorithm, as compared to Chord variants, and demonstrate sharp upper and lower bounds for both worst-case and average case settings. In addition, we theoretically analyze the hop reducing in SChord and derive the fact that SChord can significantly reduce the routing hops as compared to Chord. Several simulations are presented to evaluate the performance of the algorithm and support our analytical findings. The simulation results show the efficiency of SChord.

## 1. Introduction

The emerging applications of peer-to-peer technologies are providing users with cheap and powerful facilities for communication. Due to their decentralized nature, the peer-to-peer applications are enjoying growing popularity. Their architectures allow for wide availability of network services. Peer-to-peer networks fall into two categories, unstructured and structured networks. Structured peer-to-peer networks are appealing because they can provide decentralization, self-organization, failure resilience, and good worst-case lookup performance for applications. However, they suffer from high latencies in average cases. Since overlay connections span wide-area networks, the overall lookup time is strongly dependent on the latencies between the intermediate nodes of the lookup. It is important to minimize the hop count of the overlay network.

Chord [1] is a popular topology for structured peer-to-peer networks, where nodes are arranged in a ring. Chord methodology partitions the ring by introducing forward links so that message routes can skip consecutive nodes. These forward links roughly skip nodes in power-of-two segments around the ring and thereby make routing easy-to-implement and demonstrably efficient. The path length of routes in a Chord network with *N* nodes is bounded by ⌈log⁡*N*⌉ in the worst-case and ⌈log⁡*N*⌉/2 on average. A thorough analysis of the Chord approach can be found in [2]. Although in general the Chord approach is both interesting and worth-while, the actual implementation of the lookup protocol has some performance and reliability drawbacks.

Several methods [3–5] have been proposed to improve Chord lookup by exploiting the topological information of the underlying physical networks. Proximity neighbor selection (PNS) is one of the representative techniques that has become widely used. This seeks to select, among the possible next hops, the one that is the closest in the physical network or the one that represents a good compromise between progress in the identifier space and proximity. PNS methodology can benefit the overall latencies of the system when peer communication latencies vary significantly, but it may result in a greater number of routes or longer latency.

As an alternative to minimizing lookup latency via PNS, paper [6] describes an optimal routing scheme based on bidirectional lookup. This scheme calculates the optimal route with the least hop count by solving a coding problem based on the signed digit representations of minimal hamming weight. Although this optimal scheme can achieve ⌈log⁡*N*⌉/3 + *O*(1) hop count on average, any intermediate node failure during a lookup terminates the lookup and yields the worst-case hop count ⌈log⁡*N*⌉/2. F-Chord [7] proposes an alternative approach to reducing the hop count by producing fingers based on the Fibonacci number system. Inspired by F-Chord, paper [8] generalizes the design of the Chord finger table for fewer routing hops. The works [7, 8] improve the diameter by paying off a corresponding increase in the degree.

In this paper we propose a new splay tree-[9] based solution to arrange and maintain the finger table called SChord. Briefly, the main contributions of our work are as follows.

- A simple and complete splay tree-based SChord structure is provided in this paper. To the best of our knowledge, it is the first work to improve Chord by introducing the tree structure into the design of finger table.
- The lookup performance of splay finger table (SFT) is evaluated. Theoretical analysis demonstrates the search cost on SFT for keys under Zipf distribution approaches *O*(1), which is a notable improvement as compared to Ω(min⁡⁡{log⁡⁡*N*, *k*}) for lookup in Chord.
- We evaluate the availability of server selection in SChord and propose the server selection algorithm (SSA). Analysis shows that SSA is efficient as well as reliable even if there are a lot of more fingers in SChord node than Chord node has.
- We analyze the expected number of hops for the lookup of a random key both in Chord and SChord. Both theoretical analysis and simulation results show that SChord can significantly reduce the routing hops.

The remainder of this paper is structured as follows.  Section 2 summarizes related work. The detailed design of our splay finger table and how to construct and search for the splay finger table are presented in Section 3.  Section 4 describes the method of utilizing server selection via splay finger table. The results of experiments are presented in Section 5. Finally we present conclusions and potential future work in Section 6.

## 2. Related Works

Extensive work has been proposed to date multiobjective optimization for Chord-like DHTs. These studies fall into four categories, namely, optimizing the finger table, proximity neighbor selection, optimizing the routing algorithm, and data replication. We summarize the representative work for each of these categories.

One line of research improves routing via optimizing the finger table. Literature [7] proposes a family of routing schemes based on the Fibonacci number system, allowing to improve the maximum/average number of hops for lookups and the routing table size per node. It is shown that in F-Chord the diameter (i.e., the number of hops) is 0:72021 log *N* and the average path length is 0:39812 log *N*. Item [8] generalizes this result, showing how to construct an improved finger table when the objective is to reduce the number of hops, possibly at the expense of an increased size of the finger table. Monte Carlo simulation results show that the new proposed finger table provides superior routing performance and exhibits reduced sensitivity to failures.

Proximity routing used by recent papers in the area [3–5, 10] is suitable for distributed systems where underlying network connections incur long latencies. CFS [5] utilizes an internet coordinating system for its participating nodes and uses server selection to avoid visiting nodes with potential long latencies. This improves the overall routing performance. LPRS-Chord [11] discusses a random sampling technique to improve the lookup performance. It redesigns the nodes' communication message to glean lookup traversing latency. Recursive lookups server selection exhibits a better scaling behavior. LPRS-Chord is fast, incurs little network overhead, and requires relatively few modifications to the existing Chord. The author modifies the finger selection algorithm in order to achieve a more balanced distribution. The advantage of this proposal as compared to previous approaches is that it does not add any overhead to the basic Chord algorithm. The key concept in the above strategies is that carefully preconfigured routing message can benefit the later operation.

Paper [6] describes a pioneering optimal routing scheme based on the signed digit representations of minimal hamming weight. The optimal routing scheme makes use of both clockwise and counterclockwise fingers and works out the path with the least hop count. Analysis shows that the optimal routing scheme can reduce the average hop count to ⌈log⁡*N*⌉/3 + *O*(1) but does not help in the worst-case, that is, ⌈log⁡*N*⌉ hops. In addition, [12] brought us *O*(1)-hop lookup by issuing parallel queries.

The Beehive system [13] introduces a proactive replication framework for DHTs. The goal of this framework is to provide DHTs with *O*(1)-hop lookup performance for Zipf-like or power law and query distributions. One highlight of this study is that it presented an approach to alleviating the DHT hot spot problem through replication. In addition, utilizing this framework is independent of the underlying DHTs and therefore does not alter the DHT lookup algorithm.

## 3. Splay Finger Table

### 3.1. Overview Splay Finger Table (SFT)

SChord uses splay finger table (SFT) for routing. SFT has four attributes:

1. *ST*: the splay tree that provides insertion and lookup of the desired key;
2. Count: a counter that records of the number of nodes in *ST*;
3. *S*: the node that has the minimum ID value in *ST*;
4. *T*: the node that has the maximum ID value in *ST*.

Splay tree is a self-adjusting binary search tree. The hierarchy of the node is closely related to the access frequency which leads to efficient access of the frequently visited nodes. On an $\overset{-}{n}$-nodes splay tree, all these standard search tree operations have an average time bound of $O(\log\overset{-}{n})$ over a worst-case sequence of operations.

SFT has two functions: routing and caching. For routing, lookup can be performed on the SFT to find the closest preceding node *n*′ to the desired key. The lookup is then directed to *n*′ and the lookup continues until the desired key is found. Similarly, the key is routed through a sequence of $O(\log\overset{-}{n})$ nodes towards the destination. As with caching, when a lookup is performed on node *n*, *n* firstly searches its splay tree in SFT for the desired key. If the key is found, then lookup is done; otherwise *n* directs the lookup to some node *n*′ in its splay tree. After the lookup on *n*′ is finished, *n* inserts the pair (key, node) to its splay tree, where node is the successor of key in the SChord ring. Each node can set a quota for its splay tree known as CACHE_MAX. When a node's SFT reaches its quota, instead of being inserted into the node's splay tree, the new (key, node) pair will be dropped. The value of CACHE_MAX will be discussed in Section 4.

### 3.2. Settingup Splay Finger Table

**Figure 1:**
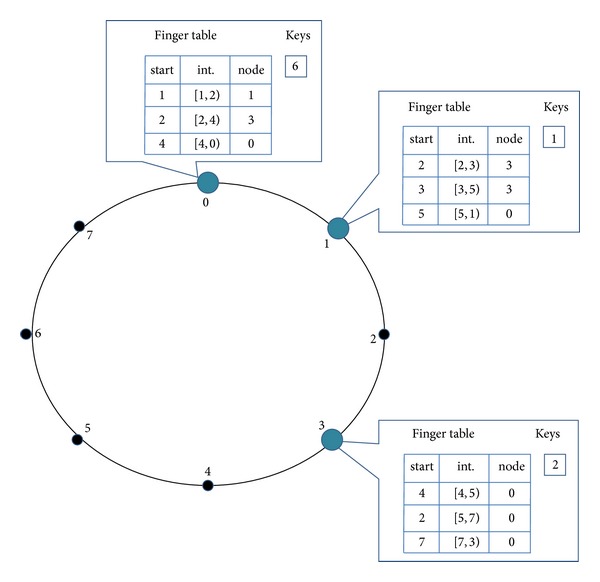
Example of Chord ring with finger tables.

The construction of SFT is similar to the construction of finger table in the original Chord. Each node maintains a SFT which contains a splay tree. When a node *n* is created, it sets up an initial splay tree for its SFT by initiating lookup RPCs to find *m* successors, that is, successor (*n* · id + 2^*i*^), 0 ≤ *i* ≤ *m* − 1. It then inserts them into SFT's splay tree. The insertion operation is the standard splay tree insertion. After the insertion, the other three attributes in SFT change accordingly.  Figure 1 depicts an example of a Chord ring with *m* = 3.

In the example above, we examine the construction of node 0's SFT step by step. When node 0 is created, all its attributes in the SFT are set to NIL. Then node 0 tries to construct its SFT by issuing a series of lookup RPCs. The fingers are then inserted to the splay tree using the splay tree insertion operation. The whole construction process is shown in Table 1.

### 3.3. Lookup on Splay Finger Table

From the previous section we learnt that the splay tree in a node's SFT covers keys only in the interval [*S* · id, *T* · id]. Thus, the lookup of key on SFT differs, according to whether the lookup key is in or out of the interval [*S* · id, *T* · id].

**Figure 2:**
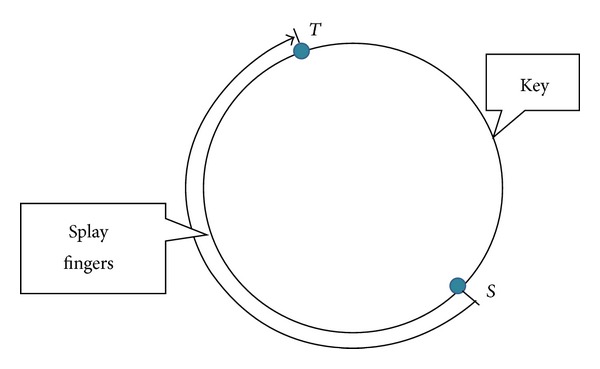
Lookup key falls out of [*S* · id, *T* · id].

In the first case as illustrated in Figure 2, when node *n* tries to resolve a lookup of key that falls out of interval [*S* · id, *T* · id], the lookup process continues on node *T*. This is because the key is on the arc $\vec{ST}$; as far as node *n* knows, *T* is the nearest node to key in identifier space; hence the node for the next hop is *T*.

The terminology which is used for describing the splay tree was introduced by Sleator and Tarjan [9]. Here we use the example in the preceding section and demonstrate how SFT changes along with the lookup process for key 1 on node 0.

**Figure 3:**
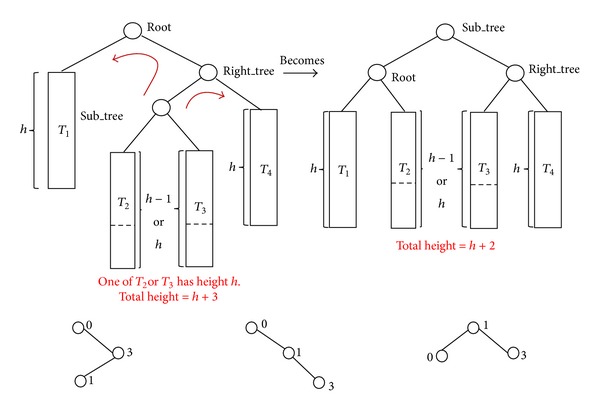
Lookup for key 1 on node 0.

Table 1 gives the ultimate state of the splay tree *ST* (Step  3) in node 0. So the lookup for key 1 has two steps; first go right then left. Such right-left access combination is called zag-zig. In the splay tree lookup operation, a zag-zig move consists of two binary tree rotations. First it rotates right at the right child of the root node, and then it rotates left at the root node, as illustrated in Figure 3.

**Figure 4:**
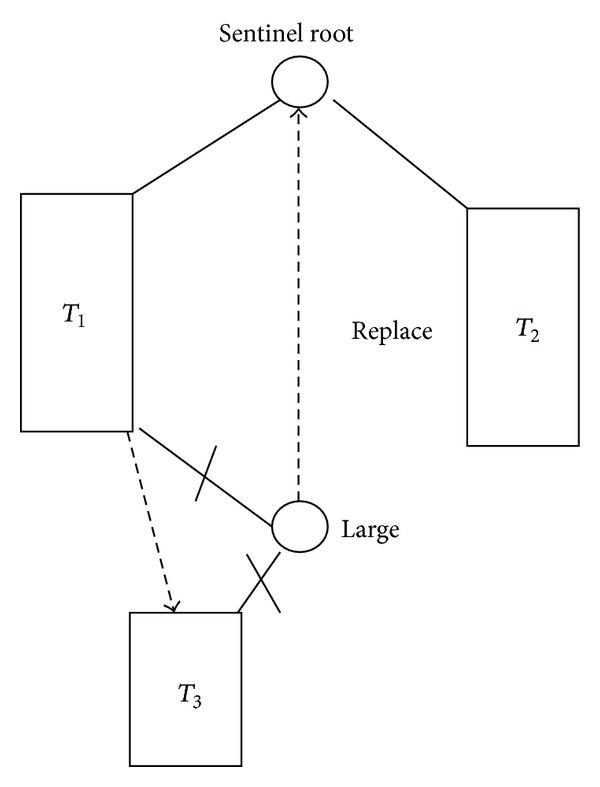
Removal of sentinel root node.

It is useful to use sentinel node pointer to replace the NIL children pointers in the leaf nodes. So after the splay search process is done and no appropriate node has been found, then the sentinel node becomes the root of the splay tree. This sentinel root node is then removed by replacing it with the largest node in its left subtree, as shown in Figure 4. After the removal, the lookup will be directed to the new root node.

When the lookup is finished, several additional postoperations are needed to update the splay tree. Since the SFT caches historical search results, for a lookup of key which does not exist in the splay tree, the key and the lookup result (key, node) pair are later inserted into the splay tree unless SFT reaches its quota, that is, CACHE_MAX. The insertion operation on splay tree consists of two steps: splay searches the key to be inserted and then replaces the root node with the (key, node) pair node if key is not found. The following is the lookup algorithm on SFT described in C-like pseudocodes; we name it slookup (see Algorithm 1).

### 3.4. Lookup Performance on Splay Finger Table

The finger table in Chord is stored in a form of sequence list or array which supports randomly access of elements. When a lookup is performed, Chord simply does a linear search on finger table by examining entry from the last to the first. Since Chord uses consistent hashing to generate nodes' identifiers, we can infer its properties from the consistent hashing. For each node *n*, the identifier difference between *n* and its successor is roughly 2^*m*^/*N*. So the number of distinct fingers in finger table is approximately *m* − log⁡_2_⁡(2^*m*^/*N*) = log⁡_2_⁡*N*. From the definition of finger table in Chord we may infer that the *i*th finger in *n*'s finger table *n* · finger[*i*], 0 ≤ *i* ≤ *m* − 1 is responsible for up to 2^*i*^ keys. The average cost of the lookup for a random key on node *n* can be calculated as follows:

(1) 2m−12m+22m−22m+···+log⁡N2m−log⁡N2m =∑i=1log⁡Ni12i=2−2N−log⁡NN=O(1),1≤N≤2m.

Thus the cost of the lookup in Chord for keys under uniform distribution is *O*(1). The cost of SFT lookup is composed of two parts: the splay tree operations, including insertion and retrieval of keys, and the update operations for SFT attributes. The SFT update operations contribute to the constant factors of the overall cost as illustrated in the pseudocode of slookup, so we only need to examine the cost of splay tree operations.

We use amortized algorithm analysis to determine the behavior of splaying over long sequences of operations. The amortized cost *a*_*i*_ is defined to be

(2) $$\begin{array}{c} a_{i}=t_{i}+c_{i}-c_{i-1}, \end{array}$$

for *i* = 1,2,…, *m*, where *t*_*i*_ is the actual cost of operation *i* and *c*_*i*_ is the credit balance after the operation *i*. So the total actual cost and total amortized cost of a sequence of *m* operations on a data structure are related by

(3) $$\begin{array}{c} \sum_{i=1}^{m}t_{i}=\left(\sum_{i=1}^{m}a_{i}\right)+c_{0}-c_{m}. \end{array}$$

Let *S*_*i*_(*x*) denote the number of nodes in the subtree rooted at node *x* after step *i* of the splaying process, then we define the rank at each step *i* of *x* to be

(4) $$\begin{array}{c} r_{i}\left(x\right)=\log S_{i}\left(x\right). \end{array}$$

We assume that, after *m* splaying steps, *x* ends up as the root. Hence we obtain that the total amortized cost is

(5) ∑i=1mai=(∑i=1m−1ai)+am≤∑i=1m−1(3ri(x)−3ri−1(x))+(1+3rm(x)−3rm−1(x))=1+3rm(x)−3r0(x)≤1+3rm(x)=1+3log⁡n−.

Thus the amortized cost of an insertion or retrieval with splaying in a binary search tree with $\overset{-}{n}$ nodes does not exceed

(6) $$\begin{array}{c} 1+3\log\overset{-}{n}. \end{array}$$

upward moves of the target node in the tree.

The total complexity of a sequence of *m* insertions or retrievals with splaying in a binary search tree that never has more than $\overset{-}{n}$ nodes does not exceed

(7) $$\begin{array}{c} m\left(1+3\log\overset{-}{n}\right)+\overset{-}{n}\log\overset{-}{n}, \end{array}$$

upward moves of a target node in the tree.

Here we recall the static optimality theorem in [9].

If every item is accessed at least once, then the total access time is

(8) $$\begin{array}{c} O\left(m+\sum_{i=1}^{\overset{-}{n}}\left(q\left(i\right)\log\left(\frac{m}{q\left(i\right)}\right)\right)\right). \end{array}$$

Consider that the nodes accessed are under Zipf distribution. We define the rank of node *i* to be *r*_*i*_, according to Zipf's law *q*(*i*) = *A*/*r*_*i*_^*α*^, where *α*  (*α* = 1) and *A* are constants that characterize the distribution. So the amortized access time becomes

(9) m+∑i=1n−(Arilog⁡(mAri)) =m+A∫1n−1xlog⁡(mAx)dx+O(1) =m+Alog⁡mAln⁡n−+A∫1n−1xlog⁡x dx+O(1) =O(m+log⁡mAln⁡n−+ln2n−),

where *A* = *θm*, 0 < *θ* < 1; thus the average access time in the worse-case is

(10) $$\begin{array}{c} O\left(\frac{\text{l}{\text{n}}^{2}\overset{-}{n}}{m}\right). \end{array}$$

In the latter section we will discuss the upper bound for $\overset{-}{n}$, that is, CACHE_MAX which is a constant. So the net potential drop over a long sequence of accesses is bound to *O*(1).

In contrast, we determine the lower bound for the lookup process in Chord when keys are under Zipf distribution. Assume that there are *k*(*k* > 0) resources with distinct ranks. Similarly, let *r*_*i*_ be the rank of the *i*th resource and let *s*_1_ *s*_2_ ⋯ *s*_*x*_ denote an arbitrary permutation of *x* of *k* integers in set {1,2,…, *k*}, for 1 ≤ *x* ≤ *k*. Then the expected cost of the Chord lookup for keys under Zipf distribution follows

(11) 1x!1∑i=1k(1/i)(∑s1s2···sx(1rs1+2rs2+···+xrsx)) =1x!1∑i=1k(1/i)((x−1)!(∑j=1xj)(∑i=1x1rsi)) =x+121∑i=1k(1/i)(∑i=1x1rsi) ≥x+12ln⁡((k+1)/(k−x+1))1+ln⁡k=Ω(x),

where *x* = min⁡⁡{*n*, *k*} and *n* is the number of distinct entries in the node's finger table. We have derived the value of *n*; that is, *n* = log⁡⁡*N*; *N* is the number of nodes in the Chord ring. Since the resources are finite and *k* is a constant, the lower bound is then Ω(*x*).

Based on the above analysis, when the lookup key is under uniform distribution, the expected cost of a search operation on splay finger table is $O(\log\overset{-}{n})$ compared to *O*(1) for the search in Chord. However, in the real world, the lookup key is nonuniform, approximately under Zipf distribution. In this case the average cost of slookup approaches *O*(1), which is a notable improvement as compared to Ω(min⁡⁡{log⁡⁡*N*, *k*}) for lookup in Chord. This is also confirmed in our experiment.

## 4. Server Selection

The use of server selection in the lookup layer strives to reduce the overall latency by choosing the node that has the least estimated lookup time (ELT) as the next hop node.

### 4.1. Background: Server Selection in Chord

When a lookup is performed, the original node *n* tries to find the predecessor of the lookup key by issuing a RPC to a node *n*′, and *n*′ then returns the closest preceding nodes in its finger table to *n*. This process is reiterated until the desired successor is found. Chord adopts this lookup method to minimize the number of necessary hops along the lookup path. Therefore the overall lookup time strongly depends on the intermediate nodes among the lookup path.

Potentially the server selection could be used at each step of the lookup process to reduce the overall lookup latency. Namely, when an intermediate node *n* tries to find the node for the next hop, instead of picking the node with the closest ID in its finger table, it estimates the time left to finish the lookup process for each finger that precedes the lookup key. So the next hop node should be the one has the least ELT.

**Figure 5:**
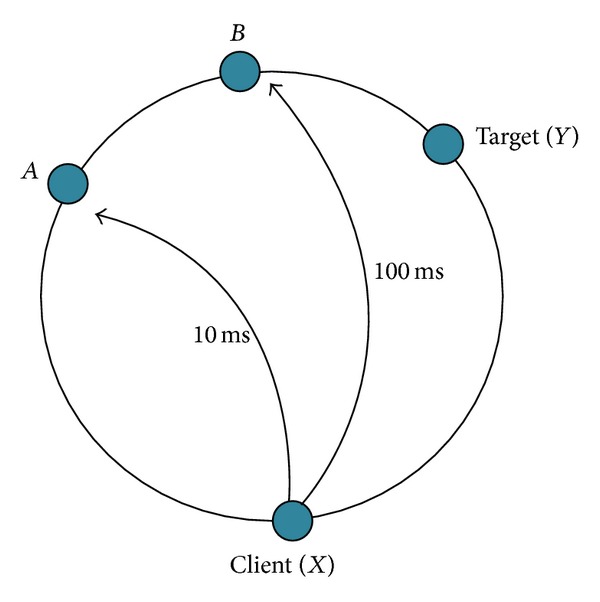
Example of choice in server selection.

Figure 5 illustrates a situation of the choice of a potentially better lookup path. When the client (node *X*) parses the lookup of target (node *Y*), it compares *Y* to its fingers and finds two matching nodes *A*, *B*. The direct one-trip time (OTT) between *XA* and *XB* could be estimated via network coordinates or querying *X*'s historical latency database. So the ELT for the rest of the lookup could be calculated as

(12) TXAY=HopLatency¯×NumHopsAY+OTTXA,TXBY=HopLatency¯×NumHopsBY+OTTXB.

It is better to pick node *A* rather than *B* if *T*_*XAY*_ < *T*_*XBY*_.

We denote by *C*(*n*_*i*_) the overall latency of the lookup for id that starts from node *n*. Then *C*(*n*_*i*_) is estimated by

(13) $$\begin{array}{c} C\left(n_{i}\right)=d_{i}+\bar{d}\times H\left(n_{i}\right). \end{array}$$

Having *d*_*i*_ be the OTT between *n* and *n*_*i*_ and $\bar{d}$ be the average per hop latency which might be obtained from the node's historical RPC latency data, *H*(*n*_*i*_) is the estimated number of hops left from *n*_*i*_ to the lookup finishes. For an *n*-node Chord ring *H*(*n*_*i*_) can be calculated as

(14) $$\begin{array}{c} H\left(n_{i}\right)=\text{ones}\left(\left(id-n_{i}\right)\gg \left(160-\log N\right)\right), \end{array}$$

where “≫” is the binary right shift operation and ones(*n*) function counts the number of significant bits of integer *n* in binary.

To utilize server selection on the original Chord, at each step of the lookup process, the node for this hop examines the fingers preceding the desired key in its finger table and chooses the one that has the least ELT as the next hop node. However, for server selection on SFT, this is a different scenario.

### 4.2. Server Selection via Splay Finger Table

Unlike the finger table in Chord, which has fixed size in fingers, the number of fingers in SFT is dynamic, varying from *m* to CACHE_MAX. So the number of predecessors of the desired key at each step of the lookup could be large. A new method needs to be discovered so that the server selection can be performed as efficiently as Chord's.

The algorithm we proposed here is inspired by probabilistic analysis. Consider the situation that at some step of a lookup on *s* node; *s* examines the predecessors in its SFT of the desired key and assumes that there are *n* of such predecessors. So *s* will then pick one of these predecessors that have a smaller ELT as the next hop node. By saying “smaller ELT,” we mean the node which has an ELT that is smaller than *t*'s ELT, where *t* is the node in SFT whose ID is the closest to the desired key. Let *p*_1_, *p*_2_,…, *p*_*n*_ be the fingers in SFT that precedes the desired key; the following gives the server selection algorithm (SSA) on SFT (see Algorithm 2).

The idea we used in SSA is to cancel the first *k* candidates, namely, *p*_1_, *p*_2_,…, *p*_*k*_, and then pick the first candidate *p*_*u*_ in *p*_*k*+1_, *p*_*k*+2_,…, *p*_*n*_ such that *C*(*p*_*u*_) is less than all *C*(*p*_*i*_), 0 < *i* ≤ *k*. If no *p*_*u*_ satisfies, then SSA will pick *p*_*n*_ by default.

We shall analyze each possible value of *k* and the probability that SSA picks the node that has the least ELT. In this case we say that SSA made the best choice. Then we will choose the best possible *k* and implement the server selection with that value. Assume that *k* is fixed for the moment. Let *M*(*j*) = min⁡_1≤*i*≤*j*_⁡{*C*(*p*_*i*_)} denote the minimum cost among the candidates 1 through *j*. Let *S* be the event in which SSA succeeds in making the best choice and let *S*_*i*_ be the event in which SSA succeeds when *p*_*i*_ is the one that has the least ELT. Since the various *S*_*i*_ are disjoint, we have Pr⁡{*S*} = ∑_*i*=1_^*n*^Pr{*S*_*i*_}. SSA never succeeds if the best one is in *p*_1_, *p*_2_,…, *p*_*k*_; we have Pr{*S*_*j*_} = 0, for *i* = 1,2,…, *k*. Thus we obtain

(15) $$\begin{array}{c} \text{Pr}\left\{S\right\}=\sum_{i=1}^{n}\text{Pr}\left\{S_{i}\right\}. \end{array}$$

In order for *S*_*i*_ to succeed, candidate *p*_*i*_ should have the least ELT; in addition, none of the candidates *p*_*k*+1_ through *p*_*i*−1_ have been chosen. Since *C*(*p*_*i*_) is under random distribution and OTTs are measured with high precision, we can tell that there are no such two candidates *p*_*v*_ and *p*_*h*_ such that *C*(*p*_*v*_) = *C*(*p*_*h*_). Hence we have

(16) $$\begin{array}{c} \text{Pr}\left\{S_{i}\right\}=\frac{k}{n\left(i-1\right)},\\ \text{Pr}\left\{S\right\}=\sum_{i=k+1}^{n}\text{Pr}\left\{S_{i}\right\}=\sum_{i=k+1}^{n}\frac{k}{n\left(i-1\right)}=\frac{k}{n}\sum_{i=k}^{n-1}\frac{1}{i}. \end{array}$$

Since the following inequalities hold for 0 < *k* ≤ *n*:

(17) $$\begin{array}{c} \int_{k}^{n}\frac{1}{x}dx\leq \sum_{i=k}^{n-1}\frac{1}{i}\leq \int_{k-1}^{n-1}\frac{1}{x}dx. \end{array}$$

The lower and upper bounds for Pr⁡{*S*} are then obtained as follows:

(18) $$\begin{array}{c} \frac{k}{n}\left(\ln n-\ln k\right)\leq \text{Pr}\left\{S\right\}\leq \frac{k}{n}\left(\ln\left(n-1\right)-\ln\left(k-1\right)\right). \end{array}$$

By differentiating the lower bound expression with respect to *k*, we obtain

(19) $$\begin{array}{c} \frac{1}{n}\left(\ln n-\ln k-1\right). \end{array}$$

Setting this derivative equal to 0, we see that the lower bound for Pr{*S*} is maximized when *k* = *n*/*e*. Thus the SSA will succeed in making the best choice with probability at least 1/*e* ≈ 0.368.

Now we determine the cost of SSA in iterations. The expected number of iterations *δ* in SSA with respect to *i* is

(20) k+∑i=1n−kik+i=n−k∑i=k+1n1i≤n−k∫k+1n+11xdx=n−kln⁡(n+1k+1).

Substitute *k* with *n*/*e*; the expression of *δ* becomes

(21) $$\begin{array}{c} n-\frac{n}{e}\ln\left(e\frac{n+1}{n+e}\right)=n-\frac{n}{e}-\ln\left({\left(1+\frac{1-e}{n+e}\right)}^{n/e}\right). \end{array}$$

When *n* grows large enough, *δ* is approximately

(22) lim⁡n→∞(n−ne−ln⁡((1+1−en+e)n/e)) =n−ne−1−ee=e−1e(n+1)≈0.632n+0.632.

Let $\hat{n}$ = 0.632*n* + 0.632. $\hat{n}$ is the sever selection cost of SSA in SChord. Then $\hat{n}<n\;\{n>2\}$. Therefore SSA is faster than server selection in Chord which calculates *C*(*p*_*i*_) for every candidate before making a choice, resulting in *n* iterations. Furthermore, by setting CACHE_MAX to (2*e*/(*e* − 1))*M* (or $\hat{n}=n$), where *M* is the bit length of the node's identifier, SSA could perform as efficient as the server selection in Chord. Better still, SSA can find the candidate that has the least ELT with probability at least 1/*e*.

### 4.3. Hop Reducing

By extending the finger table with historical lookup (key, node) pairs, at each step of the lookup, the node that resolves the lookup could potentially take larger advances towards the desired key. For example, when SFT in a node *n* is initially built, it has exactly the same fingers as the finger table does in Chord. SFT then grows with the number of lookups done on node *n* since *n* caches the historical lookup results with the corresponding keys (see Section 3). Thus for some lookup of key *k*, if *k* falls between *n* · finger[*i*] · id and *n* · finger[*i* + 1] · id, where 0 ≤ i < *m*, with some probability, there exists a cached key *k*′ in *n*'s SFT such that *k*′ ∈ (*n* · finger[*i*] · id, *k*), so *n* will choose the node in its SFT whose ID is *k*′ as next hop node, instead of *n*.finger[*i*] in Chord.

We now use (14) to determine the expected number of hops for the lookup of a random key both in Chord and SChord, respectively. We denote by *ξ* the number of hops for a lookup in Chord and *η* in SChord. The ones(*n*) function is provided as in Algorithm 3.

The cost of ones(*n*) function is determined by the number of significant bits in *n*. Assume that there are *m* of them; then the cost of ones(*n*) is *m*. Thus, for *n* ∈ [0, 2^*m*^), ones(*n*) ~ *B*(*m*, 1/2), the expected cost of ones(*n*) is *m*/2.

**Lemma 1**

Consider

(23) $$\begin{array}{c} \sum_{i=1}^{t}i2^{i}=2^{t+1}\left(t-1\right)+2. \end{array}$$

For any node *n*, let *B*_*i*_ be the event in which *n* · *finger*[*i*] is responsible for the desired key. In order for *B*_*i*_ to succeed, key should be in (*n* · *finger*[*i*] · *id*, *n* · *finger*[*i* + 1] · *id*]; hence the probability Pr {*B*_*i*_} in which *B*_*i*_ happens is

(24) $$\begin{array}{c} \text{ Pr }\left\{B_{i}\right\}=\frac{2^{i}}{2^{m}}, \end{array}$$

and the expectation for *ξ* follows

(25) Eξ=1+∑i=1m−1(i2
Pr
{Bi})=1+∑i=1m−1i2i2m+1.

Applying Lemma 1 we obtain

(26) $$\begin{array}{c} E\xi =\frac{m}{2}+\frac{1}{2^{m}}. \end{array}$$

**Lemma 2**

For a SChord node with *m* + *c* fingers, the cost of a lookup for any key never exceeds *m* + 1 − log⁡⁡*c*.

**Proof**

SChord contains all the *m* fingers borrowed from Chord and *c* cached fingers. From the property of consistent hashing, we know that the *c* cached fingers divide the ID space to *c* segments with each segment length log⁡⁡(2^*m*^/*c*) = *m* − log⁡⁡*c* in bits. After the first lookup step is done, the difference between current hop and the desired key in ID space is then less than a segment. Finally, the total number of hops, including the initially first lookup step, never exceeds *m* + 1 − log⁡⁡*c*.

Let us examine the expression of *Eξ*. By substituting *i*/2 in the expression with *m* − log⁡⁡*c*, when *m* > log⁡⁡*c* and log⁡⁡*c* ≤ *i* ≤ *m* − 1, according to Lemma 2, we obtain an upper bound for *Eη*:

(27) Eη≤1+∑i=m−log⁡cm−1m−log⁡c2m+1−i+∑i=1m−log⁡c−1i2i2m+1=m−log⁡c2(1−1c)+m−log⁡c−22c+12m+1=m−log⁡c2−1c+12m+1, m≥log⁡c+2.

So the expected number of hops *τ* reduced by slookup as compared to lookup is

(28) $$\begin{array}{c} \tau =E\xi -E\eta \geq \frac{\log c-2}{2}+\frac{1}{c}. \end{array}$$

Let *ρ* denote the percentage of the promotion; then we have

(29) $$\begin{array}{c} \rho =\frac{\tau }{E\xi } \end{array}$$

and recall the fact that CACHE_MAX = (2*e*/(*e* − 1))*M*, so *c* = CACHE_MAX − *M* = ((*e* + 1)/(*e* − 1))*M*, so we obtain a lower bound for *ρ*:

(30) $$\begin{array}{c} \rho =\frac{\tau }{E\xi }\geq \frac{\left(\log c-2\right)/2+1/c}{m/2+1/2^{m}}>\frac{6.44}{m}. \end{array}$$

Since the latency for each routing hop is relatively fixed, we may infer that when *m* = log⁡⁡*c* + 2, there are up to 1384 nodes, which is also the approximate number of peers in a popular Bit-Torrent swarm. SChord increases the lookup performance by 62% with respect to Chord. For the network of 2^24^ > 16,000,000 nodes, SChord increases the lookup performance by 26% as compared to Chord.

## 5. Experiments

In this section, we conduct experiments with the SChord by simulation. The protocol is implemented as recursive style so each intermediate node forwards a request to the next node until it reaches the successor of the desired key, and server selection is optional at each of these intermediate nodes.

### 5.1. Hop Estimation

We first consider the ability of (14) to predict the lookup hops accurately. Equation (14) is used both in the server selection and in the analysis of hop reducing. So the accuracy of (14) is crucial. We test (14) in networks consisting of nodes varying from 25 to 1000. For each network size, we perform 1000 lookups of random keys. The error *E* of the 1000 lookups is calculated as follows.

Let *p*_*i*_ be the actual number of hops for the *i*th lookup and let *q*_*i*_ be the predicted number of hops for this lookup, for *i* = 1,2,…, 1000. We have

(31) $$\begin{array}{c} E=\frac{1}{1000}\sum_{i=1}^{1000}\left|p_{i}-q_{i}\right|. \end{array}$$

**Figure 6:**
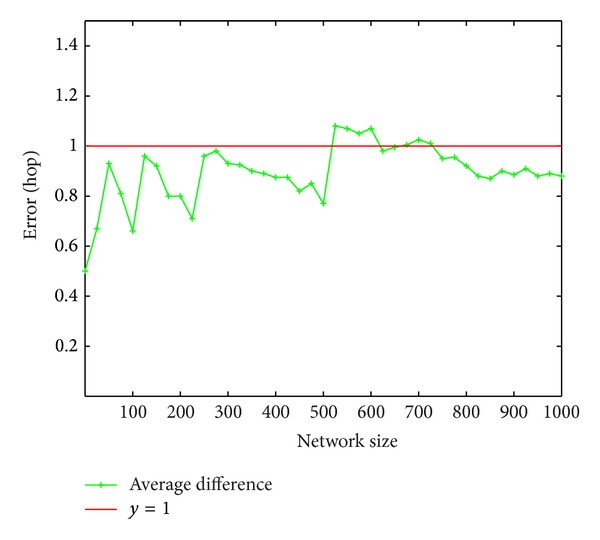
Error distribution for 1000 lookups.

Figure 6 plots the error of networks with size varying from 25 to 1000 nodes. Thus for the majority of networks the lookup errors are below one hop and for all of the networks the errors are below 1.2. We draw the conclusion that (14) is relatively accurate.

### 5.2. SFT Lookup Performance

In this experiment, we evaluate the finger table lookup performance in Chord and SFT lookup performance in SChord, respectively. In the real world, the lookup keys are under Zipf distribution. So we randomly generate a total of 125 resources ranking from 1 to 125; then the lookup keys are restrained to those 125 resources. In this case, we test the lookup time for lookups on finger table and SFT over up to 1,000,000 lookups.

**Figure 7:**
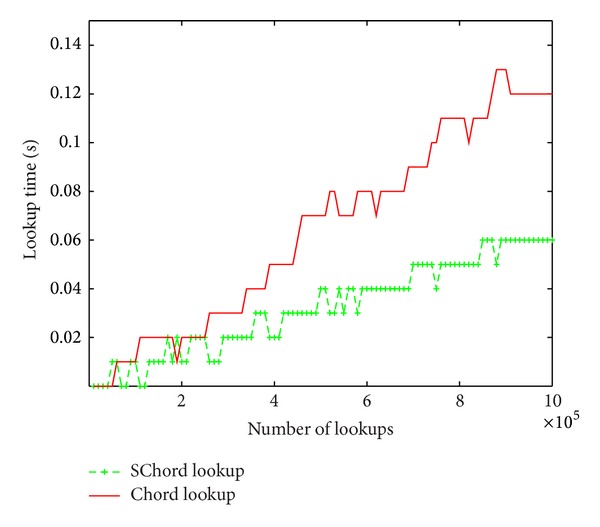
Lookup time for keys under Zipf distribution.

Figure 7 plots the lookup time curves. We can see that the SChord lookup curve is relatively steady and the Chord lookup curve grows faster. Therefore, SChord can achieve much better lookup performance than Chord on a large scale of lookups.

### 5.3. Hop reducing

We shall verify the feasibility to predict the expected number of hops involved in one single lookup or slookup. According to the previous induction we know that the expected number of hops involved in one single lookup should be *Eξ* = *m*/2 + 1/2^*m*^. This is based on the hypothesis that (14) holds for arbitrary IDs. Besides, the previous induction gives us an upper bound for the expected number of hops involved in one single slookup; that is, *Eη* ≤ (*m* − log⁡⁡*c*)/2 − 1/*c* + 1/2^*m*^ + 1. Finally *Eξ* and *Eη* are the predicted hops; we shall match them with the real hop data.

**Figure 8:**
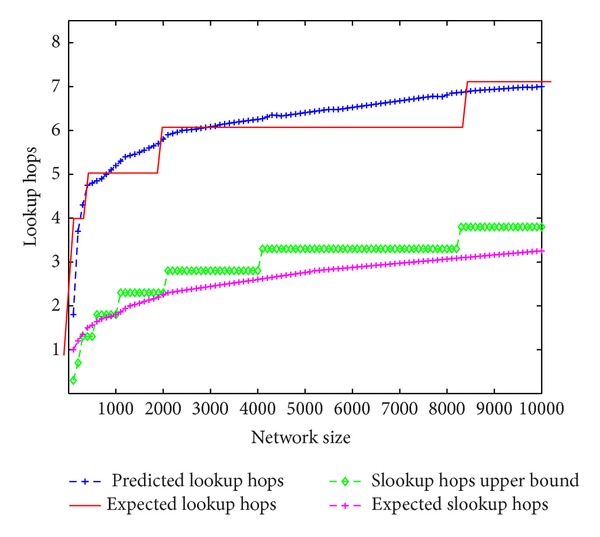
Prediction of expected lookup and slookup hops.

Figure 8 compares the experiment hop data for network size 1 through 10,000 with the values evaluated by the expressions of *Eξ* and *Eη*. For each network size, we randomly choose 10,000 nodes with each node resolving a lookup for a single random key. The figure shows that the upper bound works well when there are more than 1000 nodes in the SChord ring. In addition, the difference between estimated lookup hops and expected lookup hops is less than one hop; thus the prediction is quite accurate.

**Figure 9:**
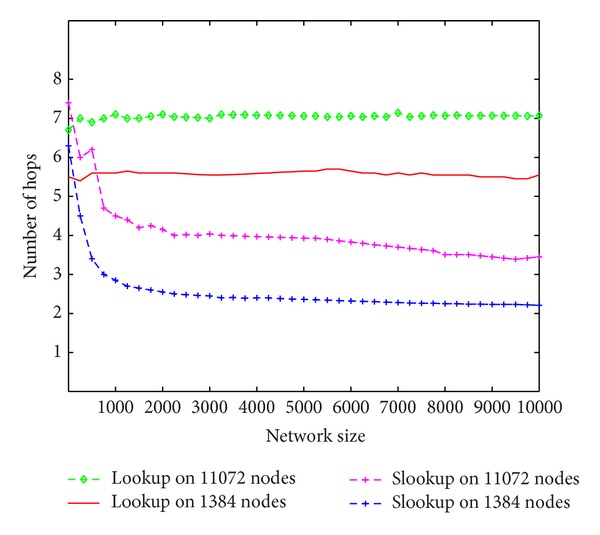
Hop reducing.

Since slookup caches the historical data, the number of fingers in a node's SFT increases with the lookups performed on this node until count reaches CACHE_MAX. Meanwhile, the expected number of hops for one lookup on this node decreases while Chord has no way of reducing hops so the expected cost of lookup is only relevant to the network scale.  Figure 9 is the comparison of the average number of hops required between lookup and slookup in two different networks of 1389 and 11072 nodes, respectively. In this experiment, for each network scale, we randomly choose a node to perform 1 through 10,000 lookups and slookups and then record the average number of hops for lookup and slookup.

**Figure 10:**
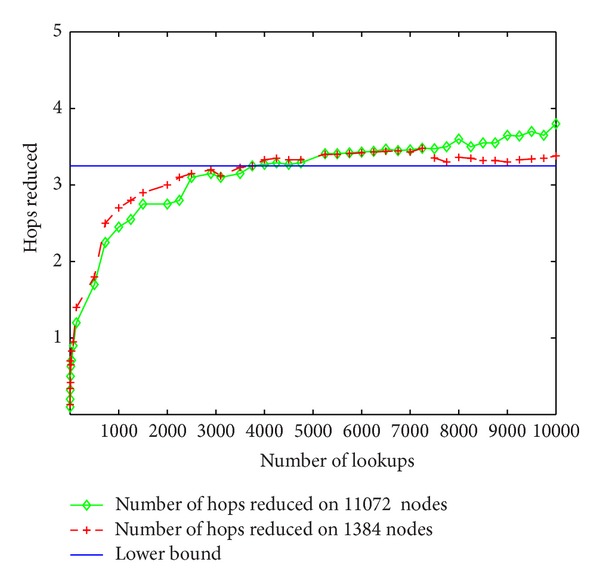
Hops reduced by SChord.

Accordingly, Figure 10 plots the number of hops reduced by slookup compared to lookup in Figure 9. The preceding induction gives a lower bound *y* = (log⁡⁡*c* − 2)/2 + 1/*c* for it. From the figure we learn that this lower bound works well after some initial slookup done on the node. In this test, the lower bound holds for around 4000 initial lookups. For systems where data are accessed in blocks or lookup operations are resolved frequently, this initial lookup requirement could be easily met.

In conclusion, the experiment results confirm that the lower bound for reducing hops is possible in practice. Hence, SChord increases the lookup operation performance by 62% at the maximum when there are 4*c* nodes in the network. Moreover, when the network size grows to as many as 16,000,000 nodes, SChord could still increase the lookup operation performance by 26% which is an immense improvement.

## 6. Conclusions

SChord is a highly scalable, available, and efficient resource location protocol. It takes advantage of splay tree where lookup caching can be used to accelerate later lookups. So the lookup performance increases based on the total number of lookups done on the node. Theoretical analysis and simulation results both confirm the fact that the lookup performance in SChord has increased up to 62% in comparison with Chord. Moreover with larger CACHE_MAX the lookup performance can be further enhanced in SChord. Detailed studies in the design space of SChord also bring interesting and useful results in building a load balance P2P system in practice.

Further study will focus on recycling mechanism for SFT so that SChord can adjust to resource change in the dynamic network circumstance.

## Figures and Tables

**Algorithm 1:**
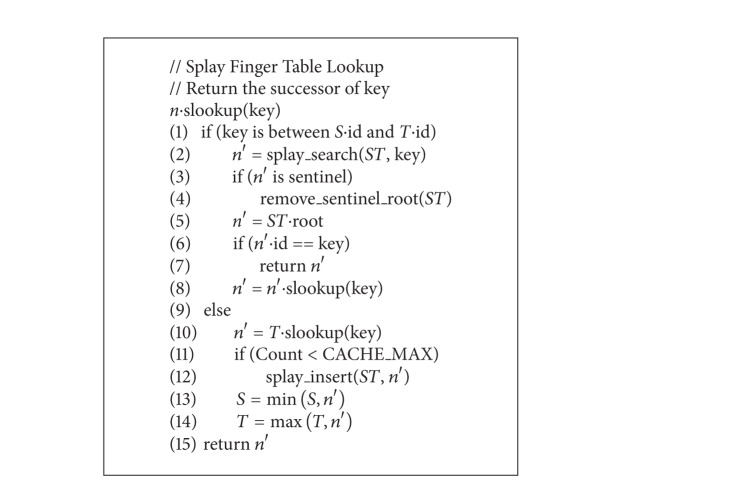


**Algorithm 2:**
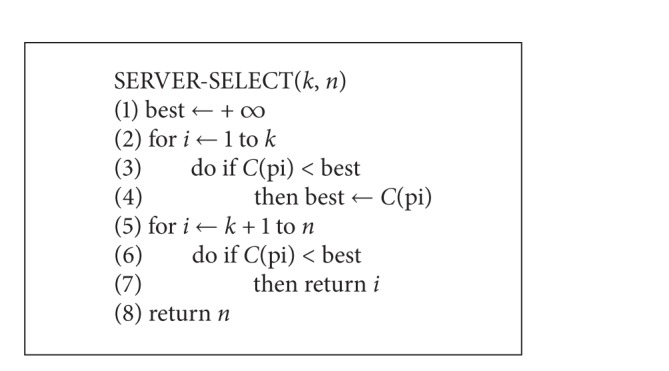


**Algorithm 3:**
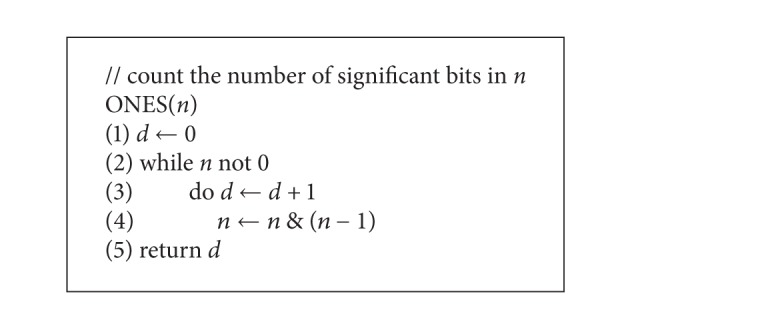


**Table 1:** Construction of node 0's splay finger table.